# Training in Dietary Practices and Physical Activity to Improve Health among South Asian Medical Students

**DOI:** 10.1155/2014/610180

**Published:** 2014-09-30

**Authors:** Divyanshi Shani, Archana Nimbalkar, Ajay Phatak, Somashekhar Nimbalkar

**Affiliations:** ^1^Department of Physiology, Pramukhswami Medical College, Karamsad, Anand, Gujarat 388325, India; ^2^Central Research Services, Charutar Arogya Mandal, Karamsad, Anand, Gujarat 388325, India; ^3^Department of Pediatrics, Pramukhswami Medical College, Karamsad, Anand, Gujarat 388325, India

## Abstract

*Introduction.* We designed a pilot intervention to test the effect of a training program on the dietary and physical activities of medical students after weekly group discussions about healthy living, maintaining a healthy diet, and healthy lifestyle. *Methods.* Two groups of students from first and second years of medical school were selected with the intervention group having high BMI (overweight or obese) while control group had normal BMI. An eight-week educational intervention was completed. A closed Facebook group ensured continuous communication. *Results.* Out of 42 participants, 19 were controls and 21 received educational training. Male : female ratio was 1 : 1.7 in control group and 1 : 1.3 in intervention group. The mean (SD) weight gain in controls (1.16 Kg, SD = 1.51) was higher than that in intervention (0.13 Kg, SD = 3.22) group (*P* = 0.2). The average reduction in caloric intake was higher in control group (117.85, SD = 258.48) vis-a-vis the intervention group (73.22, SD = 266.84) (*P* = 0.61). *Conclusion.* Educational intervention in small groups for bringing about behavioral changes towards dietary, nutrition, and physical activity can lead to changes in the target population. The short duration of our study was a limitation which should be overcome in future studies.

## 1. Introduction

Obesity is one of the leading risk factors known to be capable of causing devastating effects on various body systems contributing to 15% of the world's obese population [1]. Obesity occurring in adolescence can continue into adult life with consequences such as type 2 diabetes mellitus, hypertension, dyslipidemia, metabolic syndrome, polycystic ovarian disease, and coronary artery disease [1]. Almost 62% of the obese live in developing countries with India and China jointly contributing to 15% of the world obese population [2]. Obesity contributes significantly to the poor health of nations and places a significant economic burden on countries [3, 4].

Interventions to manage overweight and obesity have been studied with different approaches. They can be family based, community based, or school and college based interventions. A systematic review of school based interventions concluded that the interventions targeted to only diet or physical activities are not as effective in improving obesity measures as a combined intervention since it is heavily dependent on population and context [5]. Community based interventions with a school component are shown in a systematic review to be more effective than interventions targeted in the community alone [6]. Another systematic review which looked at overweight and obesity in the transition from adolescent to adult life has concluded that interventions targeted to small size groups were successful in preventing weight gain. These mostly included self-weighing and feedback for behavioral change. It recommended that large scale studies should be conducted to generate more robust evidence [7]. Most of these studies have been carried out in developed country settings and have not been tested in Indian settings.

Dietary patterns and physical activity of populations are culture specific and more local evidence needs to be generated to recommend strategies to improve obesity levels in Indian adolescents and young adults. Indian diet differs from the west and with greater western influences there seems to be a nutritional transition in India with import of the fast food culture of the western world [8]. There is scarce literature related to obesity in the Indian setting with a recent single small study conducted in North India showing success in a school based intervention [9]. A recent review of evidence of interventions for obesity that may be applied in Indian settings had very few studies from India, and it concluded that there are large gaps in knowledge about prevention and management of obesity. This review brings out the fact that there may be many interventions that are possible which need to be studied in India, while giving careful consideration to the various issues in study design such as feasibility and composition of interventions [10].

Medical professionals play an important role in the management of obesity. However, being a part of the same society these lifestyle diseases can afflict them too. Students in medical professional courses should develop healthy habits both dietary and physical as these will not only influence their own health and fitness but will also influence their counseling to patients under their care when they become physicians in due course of time [11]. These students lead a stressful lifestyle and may have unhealthy habits of diet and physical exercise which predispose them to being overweight and obese [12]. A public problem of obesity thus extends itself to the smaller subset of health professionals in the society.

Earlier work elsewhere has shown that physical activity in students is mainly driven by the desire to lose weight [13]. Additionally, physical activity levels in medical students can continue to be part of their daily routine and can enhance their ability to prescribe and support physical activity in patients that they see in practice later [14, 15]. Addressing the issues faced by future medical professionals should possibly improve their confidence in the interventions that they may be required to prescribe in the future.

There are no studies related to future medical professionals that have been published from India. We sought to address this gap in knowledge by designing a short duration educational intervention study to observe the effectiveness of small group sessions on change in the dietary and physical activity of a small group of undergraduate students in the first and second MBBS of a medical college in central Gujarat.

## 2. Methods

### 2.1. Study Design

Nonrandomized controlled trial.

### 2.2. Study Settings

Medical College attached to a tertiary care rural teaching hospital in Western India.

### 2.3. Study Procedure

A total of 200 medical students in their first and second years were invited to participate in the study. They were apprised of the need to attend group sessions before and after an eight-week period. All students underwent a physical examination before entry into college and thus there were no exclusion criteria except being underweight. Written informed consent was obtained from all the voluntary participants before recruitment. The study was approved by institutional ethics committee.

The whole body composition was measured by Tanita machine for all the participants. The students with BMI >23 were categorized as overweight, while those with BMI >25 were categorized as obese. The students having BMI less than 18 were categorized as underweight while students having BMI between 18 and 23 were considered as normal. This classification is in accordance with that for Indians at the time of the study [16]. The normal participants served as controls while the overweight/obese participants received an intervention in the form of videos related to consequences of early onset of obesity/diabesity followed by small group discussion (in a group of 5 participants) pertaining to diet control and improving physical activity. This was continued every week for eight consecutive weeks. The participants who could not attend the weekly sessions received individual counseling at a suitable time in the same week. Considering the manpower involved, the recruitment was stopped after enrolling 25 volunteers in the intervention group.

A physical activity questionnaire, a 24-hour recall questionnaire for estimating caloric intake and demographic information, was collected at the baseline and again at the end of eight weeks for all the participants. The physical activity was estimated in terms of Metabolic Equivalents (METS) using the physical activity questionnaire. The study was started during the summer season. Additionally, a closed group on Facebook was made where regular inputs were updated once a week, especially during vacations as a part of constant input and reinforcement. In the course of eight weeks the investigators read from the book* Aim for Healthy Weight* from National Heart, Lung and Blood Institute. The videos were collected from the internet, while the calculation of the total caloric intake was done from the* Textbook of Preventive and Social Medicine* by Park. The sessions conducted every week sensitized the students not only regarding advantages of dietary control and carrying out some physical activity, but also about risk factors and complications of obesity in the latter half of the life.

### 2.4. Statistical Analysis

Descriptive statistics (mean (SD), frequency (%)) were used to depict the baseline characteristics of the study population. Paired *t* test was used to determine the effect of training program on diet control and physical activity in both the groups. Independent sample* t* test was applied to determine whether the improvement was different across the groups.

## 3. Results

Out of the first 58 respondents recruited in the study, 21 were normal (control group), 26 were overweight/obese (intervention group), and 11 were underweight. All the 11 underweight respondents were excluded from the study. Three participants from intervention group and 2 participants from control group were lost to followup at various stages of the study. Total 42 participants completed the study (19 in control group and 23 in intervention group).

Of the 42 participants, 26 were studying in first year. Male to female ratio was 1 : 1.7 in control group and 1 : 1.3 in intervention group. The physical activity and caloric intake were similar in both the groups but the BMI, weight, and visceral fat were significantly less in the control group at baseline (Table 1).

The physical activity, diet control, and visceral fat proportion improved in the intervention group although the improvement was not statistically significant. Weight and BMI had a very small fluctuation that was not statistically significant.

In the control group despite apparent improvement in diet control and physical activity, a statistically significant weight gain was observed (Table 2).

The mean (SD) weight gain in controls was higher compared to intervention group although the difference was not statistically significant (1.16 (1.51) versus 0.13 (3.22), *P* = 0.2). Similarly the improvement in physical activity (METS) and visceral fat was better in intervention group as compared to control group. The mean (SD) reduction in caloric intake was higher in controls as compared to intervention group although the difference was not statistically significant (117.85 (258.49) versus 73.22 (266.84), *P* = 0.61).

## 4. Discussion

A systemic review of strategies to prevent weight gain in workplace or college settings did not show any evidence to suggest any preferable strategies to reduce weight gain. Though this systemic review included only two college based interventions and hence was not powered to find a difference, it brings out the fact that this period of life, when habits are made, has not received enough attention [17]. Present study in spite of being shorter in duration does indicate that there is no significant weight gain.

While the caloric consumption and METs are not different in the two groups, there is a difference in the weight and the visceral fat of the groups which is due to the basis of selection of groups. The eight-week intervention was expected to reduce the calories and increase the METS in the intervention group. Table 2 shows the pre- and postintervention values in the control and intervention groups. There is no difference seen in both groups except a significant weight gain and hence increase in BMI in control group over the eight-week period.  Table 3 shows a comparison of the difference in change in pre- and postintervention in the two groups which is not significant. It shows that there is a reduction in calories and visceral fat in both groups with an increase in METs. However the changes do not achieve statistical significance.

Subgroup analysis showed that females had an increase in physical activity after intervention while males had decreased caloric consumption after intervention. There was decrease in visceral fat (VF) and increased METS and decreased calorie consumption in postintervention group although it was not statistically significant. The increase in weight after intervention was associated with decreased VF and increased METS which may be due to increased muscle mass.

There were difficulties faced by students in various ways. Exams were held in the intervention duration and there were fewer options available for the hostel inmates for food in terms of high protein, low fat, and low carbohydrate diet. Reduced physical activity and increased sedentary lifestyle during exam time and frequent binging out for parties after the examination are issues that complicate the choice of lifestyle. All these factors led to increase in BMI and decrease in METS in control groups. Despite this pattern seen in control group our intervention group did well in terms of a decrease in visceral fat along with decrease in calorie consumption seen in males while a decrease in visceral fat with significant increase in physical activity in females which would not have been possible without a deliberate effort. Majority of students especially males had targeted on calorie consumption of 1400–1700 kcal while majority of students especially females had involved themselves in physical activity in some form or another like physical training, badminton, dancing, and football. There were some students who did not show any change in any of the parameters or showed increase in BMI and VF and decrease in METS.

The authors appreciate that there are inherent limitations to the study design. While two groups with similar characteristics with a larger sample size would have provided sound evidence, we used quasi-experimental design with a smaller sample size due to feasibility issues. Having a more exploratory design and a qualitative component to our study may have allowed us to determine some more indicators as to why this occurred. We believe that the study highlights important issues that need to be addressed while designing future studies in this area of research. Considering the difficulties faced in the conduct of the study described earlier, a multicentric longitudinal study with sufficient sample size and longer duration would have to be undertaken to eliminate the various confounding factors which have affected our study results.

## 5. Conclusion

Diet and physical activity both play an important role in influencing weight, BMI, and visceral fat. From our study we conclude that there is a trend towards a gender difference while reducing the visceral fat and BMI. Visceral fat responded the best with decrease in calorie consumption in males while it was more associated with increased physical activity in females. Because of small sample sizes, the differences seen in our study did not reach statistical significance. However the current study allows us to have a starting point to conduct larger studies over a longer duration to reach more definitive conclusions.

## Figures and Tables

**Table 1:** Baseline parameters of the two groups.

Baseline profile	Baseline parameters		*P* value
	Control	Intervention	
Height	162.63	165.41	0.34
Weight	53.83	72.94	<0.001
BMI	20.39	27.31	<0.001
Visceral fat	3.26	8.26	<0.001
Calories	1957.4	2013.00	0.79
METS	2.89	1.48	0.20

**Table 2:** Parameters of the two groups before and after the intervention (within group comparison).

	Control			Intervention		
	Preintervention	Postintervention	*P* value	Preintervention	Postintervention	*P* value
Weight	53.83	54.99	0.004	72.943	73.078	0.84
Body mass index	20.39	20.85	0.001	27.310	27.049	0.70
Visceral fat	3.26	3.21	0.75	8.26	7.91	0.16
Calories	1957	1839	0.08	2013	1939	0.25
METS	2.89	3.22	0.40	1.42	2.27	0.25

**Table 3:** Comparison of changes between groups (between group comparison).

Delta	Change in parameters between the groups		
	Control	Intervention	*P* value
Weight	1.16	0.13	0.21
Body mass index	0.45	−0.26	0.31
Visceral fat	−0.05	−0.35	0.31
Calories	−117.86	−73.22	0.61
METS	0.34	0.84	0.55
